# Resistance to quorum sensing inhibition spreads more slowly during host infection than antibiotic resistance

**DOI:** 10.1080/19490976.2025.2476582

**Published:** 2025-03-11

**Authors:** Tom Defoirdt

**Affiliations:** Center for Microbial Ecology and Technology (CMET), Department of Biotechnology, Ghent University, Gent, Belgium

**Keywords:** quorum sensing, quorum quenching, resistance, selection, AMR, gastrointestinal infections

## Abstract

Antibiotic resistance is a rising problem and new and sustainable strategies to combat bacterial (intestinal) infections are therefore urgently needed. One promising strategy under intense investigation is the inhibition of quorum sensing, bacterial cell-to-cell communication with small molecules. A key question with respect to the application of quorum sensing inhibition is whether it will impose selective pressure for the spread of resistance. It was recently shown that resistance to quorum sensing inhibition will spread more slowly during infection of a host than resistance to traditional antibiotics.

Current antibiotics function mainly by either killing bacteria or stopping their growth.^1^ This approach creates a strong selective pressure for bacteria to develop resistance. Resistant bacteria can survive and multiply even in the presence of antibiotics, while nonresistant (wild type) bacteria are either killed or their growth is halted. Consequently, antibiotic resistance is spreading rapidly, with clinically significant resistant bacteria often emerging just a few years after an antibiotic is first introduced.^1^ Antibiotic resistance is a rising problem; the WHO predicts 10 million deaths every year by 2050 and an economic cost exceeding $ 100 trillion if no action is taken. This makes the development of new and sustainable strategies to combat bacterial infections a critical societal challenge. One promising strategy under intense investigation is the inhibition of quorum sensing, bacterial cell-to-cell communication with small molecules. Quorum sensing systems control the virulence of various bacterial pathogens and inhibition of quorum sensing has been shown to effectively control bacterial diseases in plants, animals and humans.^2–6^ As a result, quorum sensing and quorum sensing interference in bacterial pathogens is an intensively studied field, with (according to the ISI Web of Science) currently around 700 papers published every year.

A key question with respect to the application of any new therapy is whether it will impose selective pressure for the spread of resistance. The general assumption in the field for a long time was that quorum sensing inhibition would exert minimal selective pressure, making the development of resistance unlikely.^7–10^ This was based on the observation that quorum sensing is not essential for the fitness of bacteria. We have challenged this assumption because it was based on experiments conducted in nutrient-rich synthetic growth media, where quorum sensing is indeed not essential.^11^ We argued that the situation might be different during infection of a host since quorum sensing controls virulence factor production, which enables pathogens to obtain nutrients in the host environment. Therefore, inhibition of quorum sensing in the host environment might cause selective pressure for the spread of resistance, and experiments in nutrient-rich synthetic growth media are not relevant in this respect.

Interestingly, 2 y later, the first study documenting resistance to quorum sensing-inhibiting brominated furanones, the most extensively researched quorum sensing inhibitors, was published.^12^ In that study, it was shown that the quorum sensing model pathogen *Pseudomonas aeruginosa* could develop resistance to these quorum sensing inhibitors in a synthetic medium where adenosine was the only carbon source. The growth of *P. aeruginosa* on adenosine relies on the quorum sensing-regulated intracellular enzyme nucleoside hydrolase. It has been suggested that the spread of resistance depends on whether quorum sensing affects fitness by modulating nutrient acquisition through private goods (products that only benefit the producer cell, such as nucleoside hydrolase in *P. aeruginosa*) or through public goods (products that benefit other cells in addition to the producer cell, such as extracellular protease that is essential for growth on extracellular proteins by *P. aeruginosa*). Indeed, if the impact is mainly on public goods rather than private goods, resistance to quorum sensing inhibition does not spread in *P. aeruginosa*.^13^ The reason for this is that the sensitive cells (which no longer produce public goods under quorum sensing inhibition) take advantage of the public goods produced by resistant cells, i.e. they act as cheats.^7^ As a result, the resistant cells have a lower fitness than the sensitive cells, and the resistance does not spread. A similar effect was recently documented in another quorum sensing model pathogen, *Vibrio campbellii*.^14^ Also for this pathogen, resistance to quorum sensing inhibition was found to spread on a solid synthetic medium where growth was dependent on quorum sensing-controlled public goods (i.e. extracellular protease), whereas the frequency of resistant cells even decreased on a medium where growth was dependent on quorum sensing-controlled private goods (i.e. flagellar motility). This research using synthetic media thus leads to the realization that whether or not resistance to quorum sensing inhibition will spread depends on the environment in which it is studied, and more specifically on the need for quorum sensing-controlled public goods and quorum sensing-controlled private goods. Therefore, in order to obtain a meaningful indication of what will happen with pathogens in a host, the spread of resistance should be studied in the environment where it ultimately matters: *in vivo*, during the infection of a host (as discussed in more detail in previously published review papers^15,16^). This challenge has not been addressed for any pathogen until recently.

Recently, the spread of resistance to quorum sensing inhibition in populations of the quorum sensing model pathogen *V. campbellii* was monitored during up to 35 cycles of infection of a host and transmission to a new host.^14^ Two scenarios were explored: one where resistance was initially rare (1% of the population) and another where it was already common (50% of the population). The first scenario simulated the emergence of a new mutation conferring resistance, while the second scenario represented the presence of a prevalent resistance mechanism (e.g. a broad-spectrum resistance mechanism possibly selected by prior antibiotic use). In the first scenario, resistance did not spread. However, in the second scenario, resistance did spread until it reached 100% of the population. Notably, even in the second scenario, the spread of resistance was significantly (~10-fold) slower when compared to resistance to antibiotics (i.e. kanamycin). This information is crucial for the future use of quorum sensing inhibitors as it indicates two key points: (1) resistance to quorum sensing inhibition will spread more slowly than resistance to traditional antibiotics, and (2) quorum sensing inhibitors should be used cautiously when treating infections caused by pathogens containing a broad-spectrum resistance mechanism (e.g. overexpression of a multidrug efflux pump, which can confer cross-resistance to quorum sensing inhibitors that need to enter the cell, such as brominated furanones^12^). In this context, agents that do not need to enter bacterial cells are preferable, as their efficacy will not be affected by multidrug efflux pumps. This could be achieved by targeting the signal molecules themselves in the extracellular environment (e.g. by enzymes that degrade them -for more info see LaSarre and Federle^5^ and Grandclément et al.^3^) or by targeting signal molecule receptors with a signal molecule-binding domain that is located outside of the cytoplasm. Examples of quorum sensing systems with such a receptor include the three-channel systems of vibrios,^2^ peptide quorum sensing systems in Gram-positive bacteria^10^ and indole signaling.^17^ In these cases, the quorum sensing-interfering agent does not need to enter the cells, and as a consequence, multidrug efflux pumps would not confer cross resistance.

There are a number of factors that might limit the extrapolation of the findings described above. First, the research did not use a quorum sensing inhibitor, but instead used mimic strains. One strain (the wild type) was used as a mimic of a quorum sensing inhibitor-resistant strain treated with a quorum sensing inhibitor. The other strain was a mutant that is nonresponsive to the quorum sensing molecules and it was used as a mimic of a quorum sensing inhibitor-sensitive strain treated with a quorum sensing inhibitor. This reflects an idealized scenario in which quorum sensing inhibition is 100% specific. Real quorum sensing inhibitors, in contrast, will likely not be 100% specific, and this can have an impact on the selection of resistance. Indeed, if there are side effects that have an impact on viability, then the spread of resistance will probably be intermediate between what was observed with the mimics and what was observed with antibiotics. Secondly, it is not yet clear whether the findings obtained in the *V. campbellii* - brine shrimp model can be extrapolated to any pathogen–host combination. The fact that *V. campbellii* behaved similarly to *P. aeruginosa* in synthetic growth media suggests that from the pathogen side, extrapolation is reasonable. Nevertheless, competition experiments in other pathogen–host systems would still be very instructive. It will probably not be straightforward, however, to find pathogen–host combinations in which such experiments are possible, both from a practical (labor-intensive experiments) and ethical (need for many test animals) perspective. Finally, the research was performed with gnotobiotic animals, i.e. in the absence of natural microbiota. Natural host-associated microbiota might affect the fitness of the pathogen, and this might be dependent on the functionality of its quorum sensing system. Indeed, some mechanisms bacteria use to compete with other microbes, such as the production of antibacterial compounds and type VI secretion, have been documented to be controlled by quorum sensing.^16^ As far as I know, however, the impact of quorum sensing on the fitness of bacterial pathogens in mixed microbial communities has not yet been studied and will require further research.

## Data Availability

Data sharing is not applicable to this article as no new data were created or analyzed in the paper.
